# A retrospective study of the mid-term efficacy of full-endoscopic annulus fibrosus suture following lumbar discectomy

**DOI:** 10.3389/fsurg.2022.1011746

**Published:** 2022-10-25

**Authors:** Zhenfei Wang, Sen Huang, Long Xu, Jinhui Bu, Guangpu Liu, Hui Wang, Jun Liang, Mengjiao Xia, Tao Chen, Chao Ma, Kerong Dai, Guangwang Liu

**Affiliations:** ^1^ Department of Orthopedic Surgery, XuZhou Central Hospital, XuZhou Clinical School of Xuzhou Medical University, XuZhou Central Hospital Affiliated to Nanjing University of Chinese Medicine, The Xuzhou School of Clinical Medicine of Nanjing Medical University, XuZhou Central Hospital Affiliated to Medical School of Southeast University, Xuzhou, China; ^2^ XuZhou Clinical School of Xuzhou Medical University, Xuzhou, China; ^3^ Graduate School of Bengbu Medical College, Bengbu, China; ^4^ XuZhou Central Hospital, XuZhou, China; ^5^ Department of Orthopaedic Surgery, Shanghai Ninth People’s Hospital, Shanghai Jiaotong University School of Medicine, Shanghai, China

**Keywords:** full-endoscopic discectomy, annulus fibrosus suture, lumbar discectomy, lumbar disc herniation, minimally invasive spinal surgery

## Abstract

**Aims:**

Full-endoscopic discectomy is associated with a high risk of disc reherniation due to the poor mechanical strength of the annulus fibrosus after scar healing. It is technically difficult to place a full-endoscopic annulus fibrosus suture. We designed an annulus fibrosus suture device that can be used to suture annulus defects under microendoscopy. The present study investigated the safety and feasibility of this technology.

**Patients and Methods:**

We retrospectively analyzed the outcomes of patients who underwent surgical treatment for lumbar disc herniation (LDH) from January 2018 to October 2020. We compared 40 patients with LDH treated with full-endoscopic annulus fibrosus suture following lumbar discectomy (LD + AFS group) with 42 patients treated with lumbar discectomy alone (LD group) regarding demographic data, symptoms, and recurrence and reoperation rates. Lumbar MRI and CT were performed 3 and 12 months. A 10-point visual analog scale (VAS) and the Oswestry Disability Index (ODI) was used to evaluate pain and the lumbar spine function.

**Results:**

The cohort comprised 82 patients, including 40 patients in the LD + AFS group and 42 in the LD group. All operations were successfully completed without serious complications. Reherniation occurred in no patients in the LD + AFS group and three patients in the LD group. The VAS scores for lumbar and leg pain and ODI score were significantly improved postoperatively (*p *< 0.05).

**Conclusion:**

Compared with conventional lumbar discectomy, full-endoscopic annulus fibrosus suture following full-endoscopic lumbar discectomy is a safe and effective minimally invasive technique that reduces the LDH recurrence rate.

## Introduction

The intervertebral disc(IVD) is an important component of spinal stability and consists of an annulus fibrosus surrounding the nucleus pulposus and the upper and lower cartilaginous endplates (1). When the annulus fibrosus ruptures and the intervertebral disc herniates, the integrity of the annulus fibrosus is compromised and the integrity of the segmental stability is affected (2). Therefore, an important goal in the treatment of intervertebral disc herniation must be tantamount to restore the IVD function and the stability of the motion segment.

Lumbar disc herniation (LDH) is a common disease in spine surgery, which often causes low back pain, radiating pain in the lower extremity, paresthesia and other symptoms, which seriously affect the daily life of patients. Most patients can relieve symptoms by conservative treatment such as traction, non-steroidal anti-inflammatory analgesics, and bed rest. For patients with persistent symptoms, surgery may be the better option, with lumbar discectomy providing faster and longer-lasting relief of radicular pain. In recent years, minimally invasive treatment of LDH has become a research hotspot. Due to its safety and efficacy, percutaneous endoscopic discectomy for LDH is being increasingly accepted by spine surgeons. However, lumbar discectomy is associated with recurrent disc herniation in 3%–18% of patients (3–5), and the reherniation rate is significantly higher for patients with a large annulus fibrosus defect (>6 mm) than for patients with a defect of <6 mm (6). Compared with limited discectomy, excessive discectomy leads to more serious disc degeneration and disc height loss (4, 7). In addition, the risk of reherniation is affected by age and weight (8, 9). Thus, an annulus fibrosus suture following lumbar discectomy is theoretically necessary to reduce reherniation and maintain the height of the intervertebral space. At present, it is very challenging to attain an adequate visual field to suture the annulus fibrosus due to the limited diameter of the endoscope channel (10). We developed a full-endoscopic annulus fibrosus suture device with an anchored wire rod through which the defective annulus fibrosus can be sutured visually under microendoscopy; this technique may reduce the LDH recurrence rate. The present study aimed to retrospectively compare the efficacy of full-endoscopic annulus suture with lumbar discectomy versus conventional lumbar discectomy in the treatment of LDH, and evaluate a novel minimally invasive method to repair the residual annulus fibrosus after discectomy to reduce the risk of recurrence.

## Patients and methods

### Patient selection

Patients with LDH were retrospectively divided into those who received lumbar discectomy combined with full-endoscopic suture of the annulus fibrosus (LD + AFS group) and those who received conventional lumbar discectomy (LD group). The ethics review board of Xuzhou Central Hospital approved the study. All patients provided written informed consent.

Patients who met the inclusion criteria were treated with full-endoscopic lumbar discectomy combined with annulus fibrosus suture in our department from January 2018 to June 2020. The final cohort comprised 82 patients who were followed up for 18 months.

Inclusion criteria: (1) LDH diagnosed based on CT, MRI, symptoms, and signs, with no response to conservative treatment for 6–12 weeks; (2) no significant lumbar instability in the flexion-extension position on radiography; (3) imaging revealed a soft protrusion or prolapse and no obvious calcification or ossification around the annulus fibrosis; (4) annulus fibrosus defect of <10 mm after discectomy.

Exclusion criteria: (1) spondylolisthesis or segmental lumbar instability; (2) scoliosis of >10°; (3) obvious calcification or ossification around the annulus fibrosis; (4) annulus fibrosus defect of >10 mm after discectomy or paracentral disc herniation and lateral disc herniation without calcification revealed on imaging; (5) Pfirrmann grading of disc degeneration not greater than grade IV; (6) needle insertion point >2 mm from the edge of the annulus fibrosus defect or a defect diameter of <4 mm; (7) acute local or systemic infection; (8) spinal primary tumor or metastatic tumor.

### Annulus fibrosus suture device

The annulus fibrosus suture device used in this study is a new product developed and designed on the basis of our previously authorized Chinese utility model patents (patent number: ZL 2017 2 0518470.6). The annulus fibrosus suture device has been approved by the Food and Drug Administration of Beijing, Registeration Certificate No.: Beijing Registeration Approval No. 20182040343. The anchoring device can be absorbed by the body.

### Protocol for annulus fibrosus suture placement

**Figure 1:**
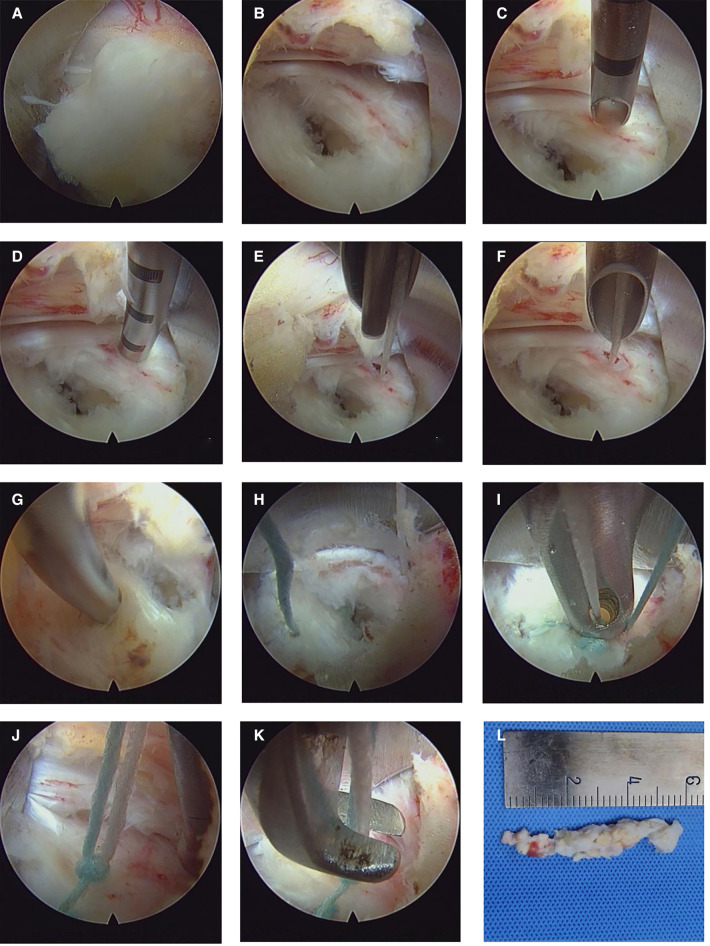
Intraoperative visualization of the annulus fibrosus suture process. (**A**) Exposure of the protruding nucleus pulposus *via* the interlaminar approach. (**B**) Excision of the nucleus pulposus reveals the damaged annulus fibrosus and an annulus fibrosus defect of about 5 mm. (**C,D**) The suture needle is inserted into the healthy annulus fibrosus 5 mm from the defect. (**E,F**) The guide-needle is used to push in the white suture with the anchoring device. (**G,H**) The same method is used to place blue sutures in the lateral healthy annulus fibrosus. (**I,J**) Placement of the knotted bilateral anchor line. (**K**) Suture cutting. (**J**) Excised nucleus pulposus.

**Figure 2:**
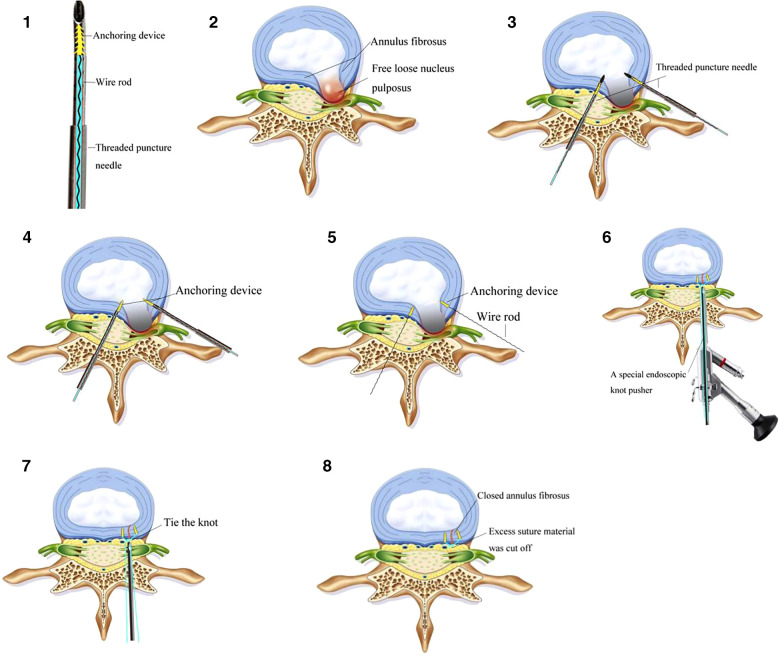
The diagrammatic drawing of the annulus fibrosus suture process. 1. The threaded puncture needle of the annulus fibrosus suture device. 2. The free loose nucleus pulposus. 3. The threaded puncture needle of the annulus fibrosus suture device was pierced into the annulus fibrosus at the bilateral breach. 4. The guidewire pushed the built-in wire rod with anchoring device into the annulus fibrosus. 5. The guidewire pushed the built-in wire rod with anchoring device into the annulus fibrosus, and the needle and guidewire were withdrawn to complete the implantation of the suture. 6–7. A special endoscopic knot pusher was used to tie the knot to repair the annulus fibrosus defect. 8. Excess suture material was cut off.

Surgery was carried out under normal local anesthesia (2% lidocaine diluted from 10 to 30 ml) with the patient in the genupectoral position. Under C-arm fluoroscopic guidance, a paramedian incision was made over the affected intervertebral space. The process of annulus fibrosus suture following lumbar discectomy was performed as follows. (1) A full endoscope with a 7.0-mm diameter working cannula entered the spinal canal. The nerve root and dorsolateral side of the dural sac were retracted. Free loose nucleus pulposus was removed with nucleus pulposus pliers after the annulus fibrosus defect and nucleus pulposus prolapse were fully exposed. The area was checked to ensure that there was no residue. A radiofrequency plasma electrode was used to shape the nucleus pulposus and annulus fibrosus, and the annulus fibrosus was fully exposed. (2) The annulus fibrosus was sutured under microendoscopy. The threaded puncture needle of the annulus fibrosus suture device was pierced into the annulus fibrosus at the side of the breach with a margin of 2–4 mm. The guidewire pushed the built-in wire rod with anchoring device into the annulus fibrosus, and the needle and guidewire were withdrawn to complete the implantation of the first suture. The other side of the suture rod was then implanted in the annulus fibrosus defect in the same way. Finally, a special endoscopic knot pusher was used to tie the knot to repair the annulus fibrosus defect. To ensure that the knot was securely tied, a surgical knot was used for the first knot, and then two square knots were continuously tied. Excess suture material was cut off after the reliability of the suture was confirmed. When the working cannula was withdrawn to the vertebral plate, the nerve root and dural sac fell back naturally, the tense nerve root relaxed, and the dural sac and nerve root resumed pulsation. The patient was then instructed to cough; if there was no obvious pain, the working cannula was safely removed. The wound was sutured and dressed with sterile auxiliary materials (Figures 1, 2).

### Patient-related outcome assessment

Visual analog scale (VAS) pain scores and Oswestry Disability Index (ODI) scores were assessed preoperatively, on postoperative day 3, and 3, 6, 12and 18 months postoperatively. Lumbar MRI and CT were performed 3 and 18 months postoperatively. The Pfirrmann grading system was used to evaluate the lumbar disc degeneration preoperatively and 18 months postoperatively.

### Statistical analysis

Quantitative data are presented as the mean and standard deviation. The independent sample *t*-test and *χ*^2^ test were used to compare data between two groups, while analysis of variance and least significant difference tests were used to compare data between multiple groups. Statistical significance was set *a priori* at *p* < 0.05. SPSS 17.0 was used for statistical analysis.

## Results

**Figure 3:**
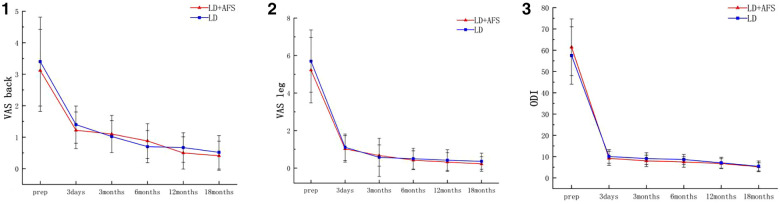
Changes in the visual analog scale (VAS) pain scores for the back (1) and leg (2), and in the Oswestry Disability Index (ODI) (3). LD + AFS: full-endoscopic annulus fibrosus suture following lumbar discectomy, AFS: annulus fibrosus suture.

**Figure 4:**
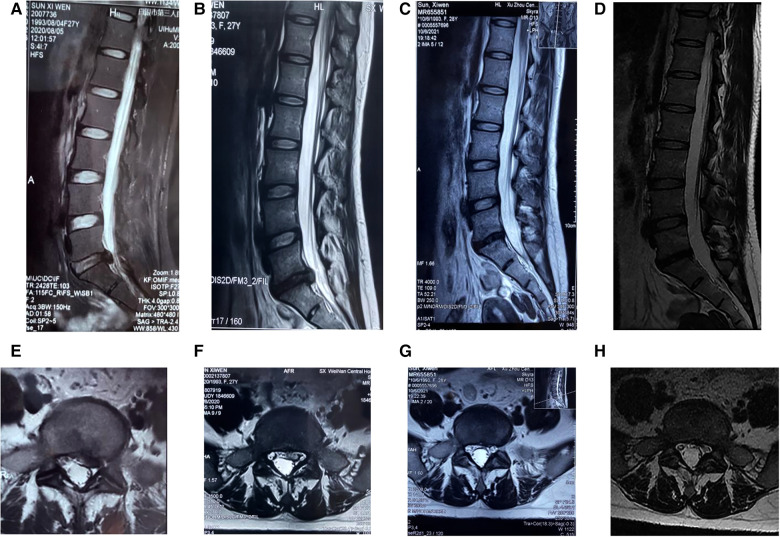
MRI of a 27-year-old woman with lumbar disc herniation with L5/S1 left center prolapse who was treated with full endoscopic nucleus pulposus resection combined with annulus fibrosus suture. (**A,E**) Preoperative sagittal and horizontal MRI showing grade III disc degeneration, compression and deformation of the dural sac, and compression of the right nerve root. (**B,F**) MRI at 3 months postoperatively shows the absence of prominent nucleus pulposus, no compression of the dura, and no obvious stenosis of the lateral recess. (**C,G**) MRI at 1 year postoperatively shows no significant disc herniation. (**D,H**) MRI at 18 months postoperatively shows the same as at 1 year.

The study cohort comprised 82 patients, including 37 females and 45 males. The average age was 37 years (range 16–59 years). There were no differences between the two groups in baseline data such as age, sex, herniated disc location, and herniation type (Table 1). There were no significant differences between groups in the preoperative VAS scores for lumbar pain and lower limb pain, and the ODI scores (Table 2 and Figure 3). The herniation was located at L3–4 in four cases, L4–5 in 42, and L5–S1 in 36. All patients underwent full-endoscopic discectomy through the interlaminar approach. All operations were completed successfully. The average operation time was significantly longer in the LD + AFS group (65.12 ± 4.56 min) than the LD group (54.45 ± 5.62 min, *p* < 0.05). The operation went smoothly in both groups, intraoperative nerve injury, dural tear, or other complications such as postoperative infection, cerebrospinal fluid leakage or aggravation of nerve root function. There was no significant difference in hospitalization days between the two groups (*p* < 0.05). Postoperative lumbar MRI showed complete resection of the herniated disc and adequate nerve decompression in all patients (Figure 4). The lumbar disc degeneration at 1 year postoperatively was assessed using the modified Pfirrmann grading system (Table 3).

**Table 1:** Baseline data.

	LD + AFS	LD	*p*-value
Gender			
M	22	23	
G	18	19	
Operation level			
L3/4	1	3	
L4/5	20	22	
L5/S1	19	17	
No. of reoperations	0	3	
Hospitalization day (Day)	7.0 ± 1.60	7.21 ± 1.77	<0.05
Operation time (min)	65.12 ± 4.56	54.45 ± 5.62	>0.05

LD + AFS, full-endoscopic annulus fibrosus suture following lumbar discectomy; AFS, annulus fibrosus suture.

**Table 2:** Pre- and postoperative VAS and ODI scores of the two groups.

Index	Time	LD + AFS	AFS	T value	*p-*value
VAS back pain	Preop	3.12 ± 1.30	3.40 ± 1.41	0.771	0.446
	Postop 3d	1.22 ± 0.58	1.40 ± 0.59	0.530	0.599
	Postop 3m	1.10 ± 0.59	1.02 ± 0.51	−0.530	0.599
	Postop 6m	0.88 ± 0.55	0.70 ± 0.51	−1.433	0.160
	Postop 12m	0.50 ± 0.51	0.67 ± 0.47	−0.443	0.660
	Postop 18m	0.41 ± 0.46	0.52 ± 0.53	−1.226	0.281
	*p*-value	<0.001	<0.001		
VAS leg pain	Preop	5.22 ± 1.75	5.70 ± 1.66	0.183	0.855
	Postop 3d	1.02 ± 0.70	1.11 ± 0.71	0.206	0.838
	Postop 3m	0.67 ± 0.57	0.57 ± 1.02	−0.940	0.352
	Postop 6m	0.42 ± 0.50	0.50 ± 0.55	−0.443	0.66
	Postop 12m	0.32 ± 0.50	0.42 ± 0.55	1.443	0.160
	Postop 18m	0.22 ± 0.39	0.36 ± 0.43	−0.526	0.781
	*p-*value	<0.001	<0.001		
ODI	Preop	61.40 ± 13.31	57.5 ± 13.49	1.589	1.120
	Postop 3d	9.22 ± 3.31	10.05 ± 3.21	−0.625	0.536
	Postop 3m	8.02 ± 2.72	9.12 ± 2.71	−1.275	0.21
	Postop 6m	7.50 ± 2.53	8.67 ± 2.39	0.206	0.838
	Postop 12m	6.75 ± 2.33	7.11 ± 2.57	0.628	0.534
	Postop 18m	5.22 ± 1.97	5.46 ± 2.53	−1.122	0.267
	*p*-value	<0.001	<0.001		

Values are presented as mean ± standard deviation. LD + AFS, full-endoscopic annulus fibrosus suture following lumbar discectomy; AFS, annulus fibrosus suture; VAS, visual analog scale; ODI, Oswestry Disability Index; Preop, preoperative; Postop, postoperative; Postop 3d, postoperative day 3; Postop 3m, 3 months postoperatively; Postop 6m, 6 months postoperatively; Postop 12m, 12 months postoperatively; Postop 18m, 18 months postoperatively.

**Table 3:** Modified Pfirrmann grade of disc degeneration in the two groups.

	LD + AFS				LD					
	I	II	III	IV	I	II	III	IV	*Χ* ^2^	*p*
Preop	0	1	23	16	0	0	27	15	0.061	0.836
Postop 18 m	4	6	17	13	2	7	20	13	0.263	0.623
*Χ* ^2^	7.633				8.241					
*p*	0.026				0.039					

LD + AFS, full-endoscopic annulus fibrosus suture following lumbar discectomy; AFS, annulus fibrosus suture; Preop, preoperative; Postop 18m, 18 months postoperatively.

## Discussion

Lumbar discectomy is a common spinal surgery. Postoperative symptomatic reherniation means that the patients experience more pain, require a more complicated reoperation, and incur additional complications and more costs (8, 9, 11, 12). A prospective multicenter randomized controlled trial that used the “Xclose” annulus fibrosus repair device to suture the annulus fibrosus defect after lumbar discectomy found that the recurrence rate of the annulus fibrosus suture group was lower than that of the unsutured group at 2 weeks and 6 months postoperatively, while the recurrence rate at 2 years postoperatively did not differ between the two groups (13). Another multicenter randomized controlled trial demonstrated that the use of a bone-anchored annular closure device to close the annulus fibrous gap after lumbar discectomy reduced symptomatic recurrence and the risk of reoperation (8). A multicenter prospective cohort study reported that the use of the “Barricaid” annulus fibrosus closure device resulted in no recurrent disc herniation and effectively maintained the height of the intervertebral disc and improved leg, back, and lumbar pain for 1 year (14). Cho et al. (15) reported that the reherniation rate after annulus fibrosus suture (3.3%) was significantly lower than that after traditional discectomy (20%); however, the relatively small sample size limited the ability to extrapolate their results to a larger population. Overall, these previous findings suggest that lumbar discectomy combined with annulus fibrosus suture has positive clinical significance.

It is difficult to suture the annulus fibrosus under full endoscopy. Li et al. (10) reported the technical points and clinical effects of annulus fibrosus suture under full endoscopy. However, in the absence of a control group, it remains unclear whether full-endoscopic annulus fibrosus suture significantly reduces the LDH recurrence rate. In our surgical experience, the limitations of the diameter of the working channel of the endoscopic system and the size of the stapler mean that non-visual annulus fibrosus suture placement carries a risk of damaging the fragile nerve roots. Therefore, our team used full endoscopy to fully visualize the annulus fibrosus defect, complete the anchoring and implantation of the first and second stitches, knot the sutures, and cut the sutures under direct vision. This process can be done using conventional endoscopy, which is widely available. In this study, the interlaminar approach was used in all patients because the translaminar approach was more intuitive than the intervertebral foraminal approach, the annulus fibrosus rupture was more clearly exposed, and it was easier to suture the annulus fibrosus under the endoscope. The 40 patients who underwent total endoscopic lumbar discectomy combined with annulus fibrosus sutures had no intraoperative adverse events, reflecting the overall safety of the operation. During the operation, the annulus fibrosus was preserved as much as possible because most of the annulus fibrosus can be repaired by itself or *via* scarring. Furthermore, less nucleus pulposus resection is conducive to maintaining the height of the intervertebral space (4, 14). However, it was unclear whether excessive resection of the nucleus pulposus reduced the recurrence of LDH. Although recurrence after discectomy is related to many factors (16), our experience suggests that the free and loose nucleus pulposus should be removed as much as possible to reduce the risk of recurrence in the short term. Annulus fibrosus sutures are mainly used to restore the integrity of the annulus fibrosus, but do not improve the disc degeneration in some patients. In addition, the remaining knot raises concerns about potential nerve irritation; however, this did not happen after surgery in the present study, There was no significant difference in ODI, VAS score of low back pain and VAS score of lower extremity pain between the two groups before and after operation. The patients in both groups obtained good clinical efficacy and the preserved knot did not show nerve irritation symptoms, possibly because of the softness of the suture material. We found that suture placement under microendoscopic visualization had a steep learning curve, and the operation time was longer in the LD + AFS group than the LD group. The longer operation time was associated with the increased time required for the suture procedure. However, we observed a gradual reduction in operation time as the proficiency with the procedure increased. During a follow-up period of 18 months, the symptomatic reherniation rate was significantly higher in the LD group (7.14%, 3/42) than the LD + AFS group (0%, 0/40). Within 6 months postoperatively, three patients in the LD group had imaging and symptomatic recurrence; two of these patients had recurrence due to weight-bearing 1 month postoperatively, which might be related to the failure of the annular fibrosus repair. Studies have shown that the mechanical strength of annulus fibrosus healing scars is still significantly lower than that of normal annulus fibrosus tissue (17). The lumbar pain, leg pain, and ODI scores were significantly improved postoperatively in both groups.

Full-endoscopic lumbar discectomy is a minimally invasive surgery that has broad appeal for both patients and surgeons. However, the technique of annulus fibrosus suture under full endoscopy is still challenging. Our novel device enables the annulus fibrosus suture to be completed under conventional small-channel endoscopy. Our study demonstrated that full-endoscopic annulus fibrosus suture is safe, reliable, and effective, reducing the risk of reprotrusion and reoperation during 18 months of follow-up. However, the present study has some limitations. Firstly, the suture technique has a long learning curve. Secondly, only a limited number of cases were followed up, and the follow-up time was relatively short. The present results require confirmation in a long-term multicenter study with a large sample size. We plan to conduct a future study with a longer follow-up duration and larger sample size.

## Data Availability

The original contributions presented in the study are included in the article/Supplementary Material, further inquiries can be directed to the corresponding author/s.
